# Validation of a Low-Burden, Once-Daily Obsessive-Compulsive Disorder Measure Over 70 Days: Ecological Momentary Assessment Study

**DOI:** 10.2196/86471

**Published:** 2026-03-30

**Authors:** Andrew Jiang, Ruibei Li, Jiaqi Fan, Adam Charles Frank

**Affiliations:** 1 Department of Psychiatry and Behavioral Sciences, Keck School of Medicine, University of Southern California, 2250 Alcazar St., Suite 2200, Los Angeles, CA, 90033, United States, 1 3234426000, 1 3234426001; 2 University of Southern California, Los Angeles, CA, United States

**Keywords:** obsessive-compulsive disorder, OCD, obsessions, compulsions, OCD symptoms, ecological momentary assessment, EMA, daily diary

## Abstract

**Background:**

Ecological momentary assessments (EMAs) are an increasingly popular tool used to measure real-time symptom burden within mental health care, including for obsessive-compulsive disorder (OCD). However, prior studies in the literature have been limited by brief assessment periods, high participant burden, and heterogeneity in both sampling and symptom assessment methodologies.

**Objective:**

This study aimed to validate a 12-item EMA questionnaire of OCD symptoms by evaluating its psychometric properties over an extended monitoring period.

**Methods:**

Adults with OCD (22/41, 53.7%) and demographically matched healthy controls (19/41, 46.3%) completed up to 70 daily smartphone-delivered EMA surveys assessing the frequency and emotional impact of obsessions and compulsions. Participants with OCD also underwent clinician-administered Yale-Brown Obsessive-Compulsive Scale evaluations at weeks 0, 2, and 10.

**Results:**

Our scale demonstrated high internal consistency (Cronbach α=0.96) and convergent validity, with significant correlation with Yale-Brown Obsessive-Compulsive Scale scores at week 2 (ρ=0.59; *P*=.004) and week 10 (ρ=0.53; *P*=.01). Participant retention (89.1%) and questionnaire completion rate (89%) were also higher than those seen in the literature (75.2% and 74.2%, respectively).

**Conclusions:**

Overall, we provide initial psychometric support for the use of a low-burden EMA tool to capture day-to-day OCD symptom fluctuations over extended periods. Such tools may enhance longitudinal symptom monitoring, improve treatment response tracking, and address limitations inherent in traditional retrospective assessments.

## Introduction

### Background

Obsessive-compulsive disorder (OCD) affects approximately 2% to 3% of the general population and is characterized by persistent, intrusive thoughts (obsessions) and repetitive behaviors or mental acts (compulsions) that cause individuals significant distress and impairment in daily functioning [1]. Traditional assessments of OCD, such as the gold-standard Yale-Brown Obsessive-Compulsive Scale (Y-BOCS) [2], ask individuals to recall symptom burden across both obsession and compulsion dimensions over the previous week and require a trained clinician to administer. Self-report assessments have also been developed and validated and include the Y-BOCS-II-SR [3], the Obsessive-Compulsive Inventory [4], and the Florida Obsessive-Compulsive Inventory [5]. These symptom scales are efficient to administer, given the self-report nature, and assess symptom severity over periods ranging from 1 week to 1 month. However, the retrospective nature of these measures has the potential to miss fluctuations in OCD symptoms over shorter timescales. These caveats to standard assessment tools highlight the need for accessible, user-friendly assessments capable of tracking OCD symptoms with greater temporal precision and reduced recall bias.

Ecological momentary assessments (EMAs), which are brief, repeated assessments delivered in real time and often sent via a smartphone in naturalistic contexts, may help fill this gap by reducing recall bias and enabling granular tracking of daily symptom burden [6]. Daily diary, a type of EMA [7], involves daily retrospective logging of experiences in lieu of in-the-moment assessments of the current state. EMAs and daily diaries both aim to minimize recall bias and maximize ecological validity, which are advantages over traditional clinical assessments [6]. In OCD research, these two approaches offer the opportunity to use technology to track day-to-day fluctuations in obsessions and compulsions in natural settings, develop person-specific modeling of symptoms [8], and consider the impact of atypical events on symptom burden [9,10].

Despite growing interest in tracking OCD symptoms using EMAs and daily diaries, prior studies have shown key limitations. One systematic review found that among 34 studies, the study periods lasted only from 1 to 7 days [7]. While allowing for OCD assessment over relatively brief intervals, this previous work did not track symptoms over more extended time spans (weeks to months). Extended monitoring is clinically meaningful, as OCD symptoms typically wax and wane across weeks to months, and improvement from treatments, including psychotherapy and pharmacotherapy, generally occurs over this timescale as well [11,12]. Still, subjective patient reports also find symptom fluctuations over shorter time frames [13], highlighting the complex nature of OCD symptoms. Additionally, current EMA protocols in the literature may be burdensome for participants, as they administer an average of 4 daily assessments and have an average dropout rate of 24.8% despite study periods lasting no longer than a week [7]. As such, these protocols may be unsustainable and impractical over longer periods, diminishing the clinical applicability of EMA. Overall, Braga et al [7] highlight the wide variation of EMA methods in all facets—from wording, scales, assessment schedules, and the like—describing the application of EMAs in tracking OCD as “fragmented” and “heterogeneous.”

### Objectives

To this end, this study—conducted as part of a larger trial—aimed to validate a novel longitudinal EMA of OCD symptoms, designed to align with the gold-standard Y-BOCS. We sought to address the limitations in study duration and compliance rate found in the literature by implementing a once-daily EMA protocol lasting 70 days in adults with OCD (n=22) and demographically matched healthy controls (n=19).

During the study, participants with OCD initiated pharmacotherapy, and Y-BOCS assessments were conducted at weeks 0, 2, and 10. We used a modified version of the scale developed by Rupp et al [8], which assessed a range of OCD symptoms and changes in response to therapy using high-frequency prompts over a short duration. Our modified version, developed in consultation with an OCD expert with decades of clinical experience, aimed to minimize participant burden to allow for a longer monitoring period that reflects a realistic treatment timeline. We examined several psychometric properties of our questionnaire—including internal consistency, discriminant validity between participants with OCD and healthy controls (HCs), and convergent validity with clinician-rated Y-BOCS scores—as well as completion rates of the entire study and compliance rates to daily EMA.

## Methods

To ensure comprehensive reporting, we adhered to the CREMAS (Checklist for Reporting Ecological Momentary Assessment Studies), an adaptation of the STROBE (Strengthening the Reporting of Observational Studies in Epidemiology) guideline. The CREMAS addresses key aspects of EMA research, including real-time data collection, participant burden, and the use of mobile technology for assessment [14].

### Participants and Recruitment 

Patients and HCs were recruited through flyers, postings on the Keck School of Medicine study recruitment website, and clinician referrals from the university’s psychiatry clinics. Eligible participants were aged 18 to 60 years. For the OCD group, inclusion required a primary OCD diagnosis, a Y-BOCS score of 16 or greater, being medication naive or off psychotropic medication for 6 months or more, being medically appropriate for pharmacotherapy, and willingness to initiate a selective serotonin reuptake inhibitor as part of standard care. HCs had no current or past OCD diagnosis. Participants’ OCD diagnosis was confirmed through the Structured Clinical Interview for *Diagnostic and Statistical Manual of Mental Disorders, Fifth Edition* (SCID-5-CV) conducted by the study psychiatrist (ACF). The Y-BOCS, a clinician-administered, semistructured interview and widely used gold-standard measure of OCD symptom severity, was used to assess symptom presence and severity [2]. The Y-BOCS includes a symptom checklist and a 10-item severity scale that rates obsessions and compulsions separately (5 items each) across core dimensions, including time occupied (frequency), interference, distress, resistance, and perceived control over symptoms.

All participants were required to be able and willing to wear a Fitbit, complete smartphone-based EMA surveys, and undergo functional magnetic resonance imaging scanning. The exclusion criteria for both groups included active substance use disorder, active suicidality, active psychosis, current psychotropic medication use, or a primary psychiatric diagnosis other than OCD. Comorbid psychiatric diagnoses were permitted in the OCD group if OCD was the primary diagnosis.

### Study Design and Procedure

The study comprised 70 consecutive days of monitoring, including weekdays and weekends. Clinical assessments were conducted at baseline (week 0), week 2, and week 10. During study onboarding, participants were trained on questionnaire completion and study technology. Surveys were administered via Qualtrics and sent once daily at a random time between 8 AM and 8 PM via SMS text messaging to participants’ personal smartphones. This random sampling approach was used to assess symptoms throughout the day (rather than at a single daily time point) and to gain additional information about participant context, as other questions—not reported herein—asked about location and activities immediately prior to survey completion. As part of a larger project, participants were instructed to wear a Fitbit Charge 5 smartwatch continuously throughout the study period to collect biometric data. Y-BOCS scores were assessed by the same study psychiatrist (ACF) for all participants at all time points.

Participants were given 3 hours to complete their EMA survey upon receiving it via SMS text messaging. Specifically, survey links expired after this 3-hour window, and participants were no longer able to submit survey responses for that day. To enhance ongoing participation in EMA completion over the course of 10 weeks, compensation was tied to maintaining weekly goals, and this compensation structure was modeled on other longitudinal EMA-based studies [15]. Specifically, weekly compensation (US $25 per week) was provided if participants completed 80% of daily EMAs (eg, 6 of 7 daily surveys per week) and wore their Fitbit 80% of the time each week. All data from all study participants who completed the 10-week study were included in this analysis, whether a participant met the 80% threshold for a given week. Finally, participants who met predetermined study withdrawal criteria were not included in data analysis. Specifically, lack of completion of at least 80% of the study questionnaires for 3 or more weeks and lack of wearing the Fitbit 80% of the time for 3 or more weeks met the withdrawal criteria.

### Questionnaire Measures

Each daily questionnaire contained 21 items assessing biopsychosocial domains, such as OCD-related anxiety, physical activity, alcohol consumption, sleep, and social interactions. Items were assessed as state-based (“currently”) or retrospective (“since the last survey”). A total of 12 items specifically assessed OCD symptoms (Textbox 1). Participants rated the frequency of obsessions and compulsions on a visual analog scale, which allowed for decimal responses. Those associated with emotions (questions 2-6 and questions 8-12) were rated on a 5-point Likert scale.

Textbox 1. **Items on the ecological momentary assessment measuring obsessive-compulsive disorder symptoms.**

- Question 1: Since the last survey, how frequently have you experienced obsessions? (0-100 visual analog scale [VAS]; skip logic applied, question 2-question 6 only appears if question 1>0)
- Since the last survey, in relation to obsessions, to what extent have you felt the following (1-5 Likert scale):
  - Question 2: Anxiety
  - Question 3: Guilt
  - Question 4: Helplessness
  - Question 5: Disgust
  - Question 6: Burdened
- Question 7: Since the last survey, how frequently have you experienced compulsions? (0-100 VAS; skip logic applied, question 8-question 12 only appears if question 7>0)
- Since the last survey, in relation to compulsions, to what extent have you felt the following (1-5 Likert scale):
  - Question 8: Anxiety
  - Question 9: Guilt
  - Question 10: Helplessness
  - Question 11: Disgust
  - Question 12: Burdened

The OCD-specific items were adapted from Rupp et al [8], who examined the treatment sensitivity of EMAs for OCD. Items were modified for compatibility with a once-daily schedule and to minimize participant burden. Study participants were trained on the distinction between obsessions and compulsions during the onboarding evaluation by the study psychiatrist (ACF) in the context of administration of the Y-BOCS.

### Data Analysis

All statistical analyses were conducted in R (version 2024.09.0+375). Internal consistency (Cronbach α) was calculated using the “psych” package (Multimedia Appendix 1). Multilevel confirmatory factor analysis (CFA) was conducted using MPlus (version 9.0) following the CFA reliability framework provided by Geldhof et al [16] (Multimedia Appendix 1).

We adopted a composite scoring approach for our EMA scale to reduce measurement noise and place all items on a single, interpretable scale of OCD symptom severity [17,18]. This approach aligns the EMA scale to the clinical measure (Y-BOCS), which is expressed as a total severity score. We evaluated internal consistency using Cronbach α to justify aggregating items and computed α separately for obsession items, compulsion items, and the combined total item set. We estimated α at two levels: (1) prompt-level α, derived from pooling all item responses on a day-to-day level, and (2) person-level α, calculated across participants after averaging items within participants across all study days. As a complementary check on reliability, we also fit 2-level CFA with a single latent factor to estimate reliability at the within- and between-person levels for the obsession items, compulsion items, and the combined total item set. We considered reliability estimates of 0.70 or greater as acceptable internal consistency.

As frequency and emotion items were scored on different metrics, we rescaled the frequency scores to match the emotion scores by dividing by 20. As the visual analog scale frequency items allowed decimal responses, the rescaled score retained fractional precision, and composite scores were derived from these unrounded values.

We then tested whether the composite EMA score discriminated participants with OCD from control participants. As the EMA uses skip logic at the frequency questions (follow-up emotion items are zero when participants have not experienced any obsessions or compulsions), we anticipated a zero-predominant distribution among control participants. As such, for each participant, we calculated a person-level proportion of symptomatic days by taking the number of days where a composite EMA score was greater than 0 and dividing it by the number of total observed days. We then compared OCD versus control on this proportion using the Mann-Whitney *U* test.

Finally, we tested for convergence between clinician-rated Y-BOCS, administered at weeks 2 and 10, and the composite EMA score by correlating Y-BOCS total, obsessions subsection, and compulsions subsection scores with their EMA counterparts, calculated as a 7-day trailing average (up to and including the day of assessment) at week 2 (approximately day 15) and week 10 (approximately day 70). Specifically, we paired the Y-BOCS total with the EMA total composite, Y-BOCS obsessions with the EMA obsessions composite, and Y-BOCS compulsions with the EMA compulsions composite and estimated Spearman rank correlations for each pairing. To account for days with missing data, days within the 7-day assessment window did not have to be consecutive.

### Ethical Considerations

This study was conducted in accordance with the University of Southern California Institutional Review Board approval (study ID IRB HS-21‐00835). Participants completed informed consent prior to study enrollment. Included in this consent was the ability for participants to opt out of study participation at any time. Data contained herein were anonymized and no participant identifying information is present. For their participation, participants received up to US $350 depending on their adherence to study protocols and were allowed to keep the Fitbit device.

## Results

### Participant Demographics

Descriptive statistics of participant demographics at baseline are presented in Table 1. The examination of baseline Y-BOCS revealed substantially higher scores for the OCD group than HCs (*t*_39_=27.55, 95% CI 23.12-26.79; *P*<.001). Age did not differ significantly between groups (*t*_39_=–0.06, 95% CI –2.91 to 2.73; *P*=.95). Gender distribution was also comparable (*χ*²_2_=1.0; *P*=.60). However, there was a significant difference in racial distribution across groups (*χ*²_12_=23.2; *P*=.03).

**Table 1.** Participant demographics and baseline assessments.

Variables^a^	Participants with OCD^b^ (n=22)	Healthy controls (n=19)	*P* value
Age (y), mean (SD)	26.8 (4.9)	26.3 (4.1)	.95
Y-BOCS^c^ total score, mean (SD)	24.9 (4.5)	0.0 (0.0)	<.001
Gender, n (%)			.60
Man	10 (45.5)	8 (42.1)	
Woman	11 (50)	11 (57.9)	
Nonbinary	1 (4.5)	0 (0)	
Race, n (%)			.03
White	8 (36.4)	2 (10.5)	
Black	2 (9.1)	2 (10.5)	
East Asian	2 (9.1)	4 (21.0)	
Indian	1 (4.5)	5 (26.3)	
Southeast Asian	2 (9.1)	0 (0)	
Hispanic or Latino	2 (9.1)	0 (0)	
More than one	5 (22.7)	5 (26.3)	
Prefer not to say	0 (0)	1 (5.3)	

a Group comparisons used independent *t* tests for continuous variables (Y-BOCS and age) and chi-square tests for categorical variables (gender and race). Significant differences were found for Y-BOCS and race; age and gender did not differ between OCD and control groups.

b OCD: obsessive-compulsive disorder.

c Y-BOCS: Yale-Brown Obsessive-Compulsive Scale.

### Compliance

**Figure 1.**
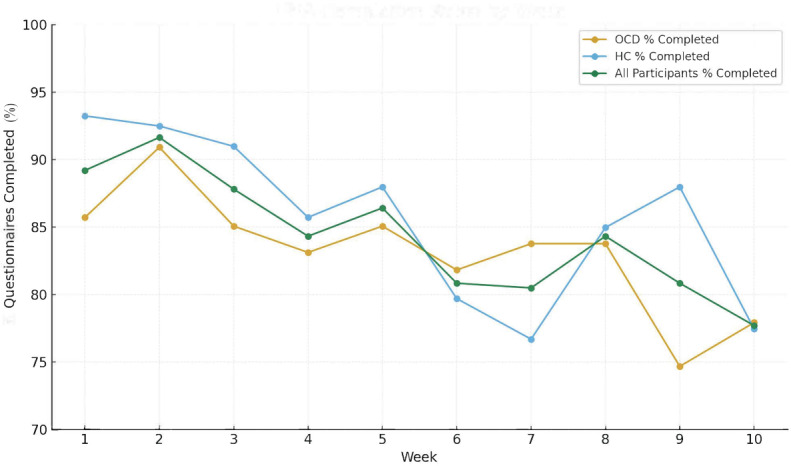
Ecological momentary assessment (EMA) completion rates by week. All participants’ percentage completed represents the mean of the obsessive-compulsive disorder (OCD) and healthy control (HC) groups by week.

A total of 25 participants with OCD and 22 participants serving as HCs were initially enrolled. Three (12%) participants with OCD and 2 (9.1%) HC participants were withdrawn from the study, given the lack of continued participation following predefined withdrawal criteria. Two (66.7%) of the 3 participants with OCD who were withdrawn from the study stopped answering all EMA questions and responding to study personnel inquiry after 3 weeks in the study. The remaining participant with OCD was withdrawn, given the lack of EMA completion and Fitbit weekly adherence for 6 weeks. One (50%) HC participant was withdrawn from the study after they stopped answering EMA questions and responding to study personnel inquiry after 2 weeks; the other HC participant was withdrawn following a lack of EMA completion and response to study personnel after 6 weeks, with low response rates for both participants during their time in the study. For all completed surveys, we report a median of 143 (IQR 100-230) seconds to complete the survey. In addition to the individual weeks in Figure 1, the overall questionnaire completion rates were 86.7%, 91.7%, and 89% among participants with OCD, HCs, and all participants, respectively. Within the OCD group, baseline Y-BOCS did not correlate significantly with the EMA compliance rate (ρ=−0.21; *P*=.34).

### Internal Consistency

The 12-item scale demonstrated high internal consistency on a prompt level (Cronbach α=0.94, 95% CI 0.94-0.95) and person level (Cronbach α=.96; 95% CI 0.93–0.98). Subscale internal consistency was similarly high: the obsessions 6-item subscale showed α=.90 (95% CI 0.89-0.91) at the prompt level and α=.89 (95% CI 0.84-0.94) at the person level; the compulsions 6-item subscale showed α=.90 (95% CI 0.89-0.91) at the prompt level and α=.89 (95% CI 0.83-0.94) at the person level. Consistent with these high alpha estimates, 2-level CFA reliability also indicated strong within-person reliability for the total scale (ω=0.94) and obsessions (ω=0.91) and compulsions (ω=0.91) subscales. Although the CFA returned high between-person estimates, the model was unstable at this level due to the small number of clusters relative to between-person parameters. We therefore emphasize within-person CFA reliability estimates alongside the Cronbach α values. Given high internal consistency, we retained all 3 composites for analysis and proceeded in calculating 3 consolidated values: a composite obsessions, compulsions, and OCD score.

### Known-Groups Validity

**Figure 2.**
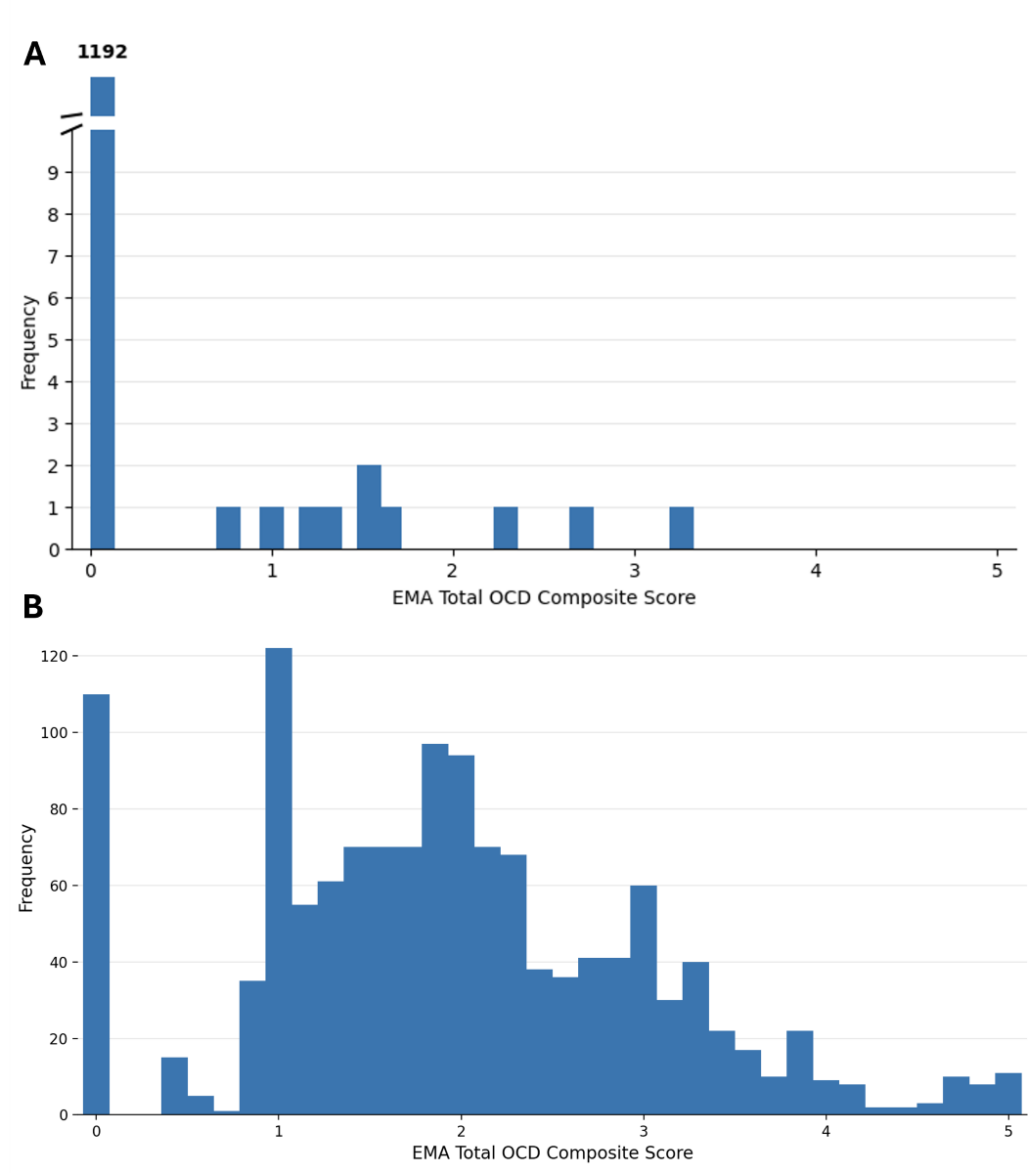
Distribution of the ecological momentary assessment (EMA) total obsessive-compulsive disorder (OCD) composite score by group. (A) Healthy controls (n=1202 person-days): 1192 (99.2%) days had a composite score of 0 (mean 0.014, SD 0.171). (B) Participants with OCD (n=1333 person-days; mean 2.03, SD 1.07).

HCs were almost universally at floor with 1192 (99.2%) of 1202 days having a composite score of 0 (Figure 2A). In contrast, the OCD group showed a broad distribution of EMA scores with a mean of 2.03 (SD 1.07) on the 0- to 5-point scale (Figure 2B). The OCD group had significantly higher composite scores than the control group (Mann-Whitney *U*=0; Z=−5.67; *P*<.001), with a large effect size (rank-biserial correlation *r*=0.91).

### Convergent Validity

**Figure 3.**
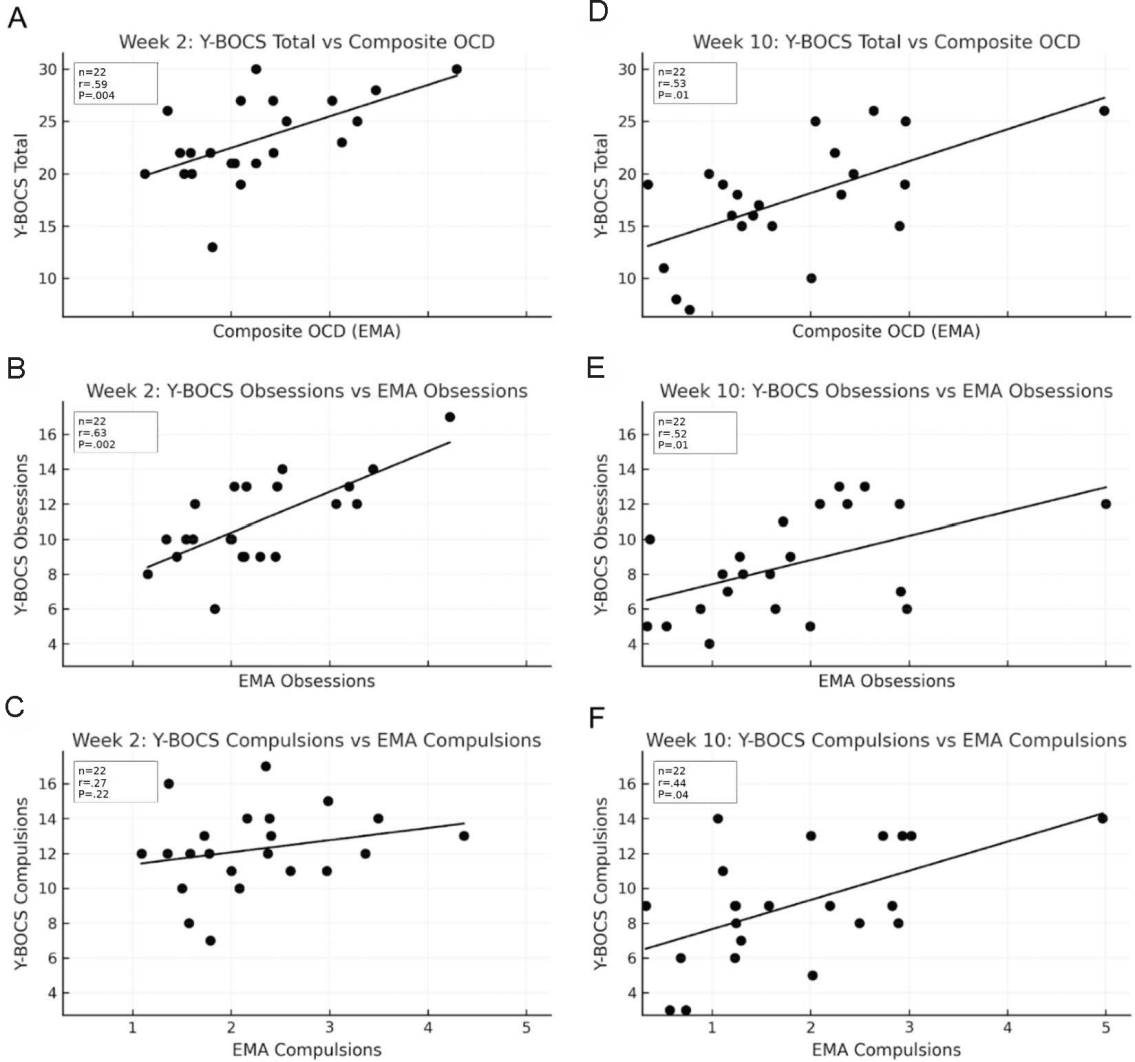
Spearman correlations between the Yale-Brown Obsessive-Compulsive Scale (Y-BOCS) and ecological momentary assessment (EMA)–derived trailing composite obsessive-compulsive disorder (OCD) scores. At week 2, it is separated by (A) EMA composite OCD, (C) EMA obsessions, and (E) EMA compulsions; at week 10, it is separated by (B) EMA composite OCD, (D) EMA obsessions, and (F) EMA compulsions. Correlations were moderate to strong for total OCD and obsessions composite and smaller for compulsions composite (week 2 compulsions not significant).

Spearman ρ indicated moderate to strong correlation between the 12-item EMA total OCD composite and the Y-BOCS total at week 2 (ρ=0.59; *P*=.004; Figure 3A) and week 10 (ρ=0.53; *P*=.01; Figure 3B). The EMA obsessions subscale showed similarly robust associations with the Y-BOCS obsessions subscale (week 2: ρ=0.63; *P*=.002; Figure 3C; and week 10: ρ=0.52, *P*=.01; Figure 3D). In contrast, correlations with the Y-BOCS compulsions subscale were smaller (week 2: ρ=0.27; *P*=.23; Figure 3E; and week 10: ρ=0.44; *P*=.04; Figure 3F), and the week 2 association was not significant.

## Discussion

### Summary of Findings

In this study, we evaluated the feasibility and validity of a once-daily, longitudinal EMA protocol for monitoring OCD symptoms in naturalistic settings over extended periods (several months). Our EMA-derived OCD symptom scale demonstrated high internal consistency, known-groups validity, and convergent validity with clinician-rated Y-BOCS scores. The EMA total OCD composite score clearly distinguished participants with OCD from HCs, and EMA-derived obsession scores showed strong correlations with clinician-rated obsession severity at multiple time points. Together, these findings support the use of daily EMAs as a reliable method for capturing OCD symptom severity and fluctuations over time.

### Connection to Prior Literature

OCD is a heterogeneous and dynamic disorder, characterized by day-to-day fluctuations in symptom severity [13]. Given this pronounced variability, there is a clear need for longitudinal symptom monitoring in real-world contexts. However, as emphasized by Braga et al [7], the existing EMA literature in OCD has been “scattered” in methods and EMA administration, with wide variation in survey frequency, question content, technological platforms, and study duration, limiting cross-study comparability. Moreover, most EMA-based OCD studies have been short, typically lasting only 1 to 7 days, restricting their ability to capture longer-term symptom trajectories, such as those in the context of a course of treatment.

Our implementation of a 70-day observation period provided a substantially longer timescale to detect both short-term symptom fluctuations and longer-term trends. We used a consistent, once-daily EMA protocol delivered via SMS text messaging to participants’ smartphones, minimizing participant burden. This design contributed to a low attrition rate of 10.9%—well below the 24.8% average reported in prior EMA studies of OCD—as well as high response rates, averaging 89.0% compared with 74.2% reported in the literature [7]. This combination of extended duration, high retention, and high response rates allowed for practical and reliable symptom tracking over time.

EMA-derived obsession scores demonstrated strong and statistically significant correlations with Y-BOCS obsessions subscale scores at both week 2 and week 10. In contrast, EMA-derived compulsion scores did not correlate significantly with the Y-BOCS compulsions subscale at week 2, although correlations were stronger and more consistent by week 10. This pattern is consistent with multiple prior studies: Cox et al [19] observed stronger associations between EMA measures and Y-BOCS obsession scores than compulsion scores. Herman and Koran [20] used a modified, self-report version of the Y-BOCS administered via handheld computer in naturalistic settings and found weaker correlations to clinician-administered Y-BOCS compulsion scores, although correlations were low for both obsession and compulsion scales in that study.

Several factors may explain the initially weaker correspondence between EMA and Y-BOCS compulsion scores. During Y-BOCS administration, clinicians can educate participants on the definition of compulsions and assist in distinguishing them from obsessions and other symptoms, potentially contributing to careful partition of symptom scores within these domains. In contrast, during EMA completion, participants may not consistently separate compulsions from obsessions. The dynamic clinical course of the sample may have also influenced our findings. Many participants initiated Selective Serotonin Reuptake Inhibitor treatment during the study period, and changes in OCD symptom burden may influence the self-report of symptoms in different domains. The lack of significant compulsion correlations at week 2 may therefore reflect transient variability or unstable symptom reporting early in treatment, whereas stabilization by week 10 may have contributed to stronger and more reliable correspondence between EMAs and clinician-rated measures.

### Limitations

Despite these strengths, several limitations warrant consideration. First, the OCD sample size was relatively small (n=22), which limited our ability to evaluate the latent structure of our EMA scale using exploratory factor analysis. In addition, the small number of participants precluded meaningful evaluation of between-person effects. The modest sample size should be interpreted in the context of stringent inclusion criteria and the demands of the parent study. Participants were required to have a primary OCD diagnosis, be medication naive or off psychotropic treatment for at least 6 months, and be willing to initiate pharmacotherapy, narrowing the eligible recruitment pool. In addition, this EMA validation was embedded within a larger multimodal study requiring near-continuous use of a Fitbit over 70 days alongside daily smartphone-based assessments, a combined burden that likely further constrained enrollment despite excellent retention and compliance among enrolled participants.

Second, participants were younger on average (mean age approximately 26.8, SD 4.9 years). As OCD presentation can vary by age, including differences in symptom dimensions, severity, and comorbidity patterns [21], future studies should validate these findings in larger and more age-diverse cohorts.

Third, EMA questions required participants to retrospectively summarize symptoms since the prior survey. Although this approach reduced participant burden, retrospective recall—even over short intervals—may introduce bias relative to in-the-moment assessments. The questions were designed to align with prior EMA studies and with Y-BOCS symptom domains, which necessitated some degree of retrospective reporting.

Finally, participants initiated pharmacotherapy during the study as clinically indicated, introducing additional variability in symptom trajectories. We also did not systematically account for acute life events or stressors that may have influenced symptom severity in the days preceding clinical assessments. Such state-dependent factors may have contributed to variability in correlations between EMA and clinician ratings. Additionally, clinician assessments were conducted only at weeks 0, 2, and 10, limiting validation of EMA measures to periods with temporal overlap.

### Conclusions

Overall, this study demonstrates that daily EMAs of OCD symptom severity correlate well with clinician-based measures while offering substantially greater temporal resolution. EMA approaches may provide clinicians and researchers with valuable insights into day-to-day symptom fluctuations, treatment response, and individualized symptom patterns that are not captured by traditional retrospective assessments. As digital mental health tools continue to evolve, longitudinal EMA protocols may play an increasingly important role in both clinical care and research on dynamic psychiatric disorders, such as OCD.

## Supplementary material

10.2196/86471 Multimedia Appendix 1 Cronbach α analysis and confirmatory factor analysis.

10.2196/86471 Checklist 1 CREMAS checklist.

## Abbreviations

CFA: confirmatory factor analysis
CREMAS: Checklist for Reporting Ecological Momentary Assessment Studies
EMA: ecological momentary assessment
HC: healthy control
OCD: obsessive-compulsive disorder
STROBE: Strengthening the Reporting of Observational Studies in Epidemiology
Y-BOCS: Yale-Brown Obsessive-Compulsive Scale
