# Supplementary material for: Validation of a Low-Burden, Once-Daily Obsessive-Compulsive Disorder Measure Over 70 Days: Ecological Momentary Assessment Study
Source: JMIR Form Res. 2026 Mar 30;10:e86471. doi: 10.2196/86471 (PMC13035074; doi:10.2196/86471)
Supplement: Multimedia Appendix 1 [file formative-v10-e86471-s001.docx]

##### **Cronbach Alpha Analysis on All Questions (average)**

Reliability analysis

Call: psych::alpha(x = participant_means %>% select(all_of(all_items)),

na.rm = TRUE)

raw_alpha std.alpha G6(smc) average_r S/N ase mean sd median_r

0.96 0.96 1 0.67 24 0.014 2.2 0.73 0.69

95% confidence boundaries

lower alpha upper

Feldt 0.93 0.96 0.98

Duhachek 0.93 0.96 0.98

Reliability if an item is dropped:

raw_alpha std.alpha G6(smc) average_r S/N alpha se var.r med.r

Q16.Obsession.frq_1 0.96 0.96 0.99 0.68 23 0.014 0.035 0.69

Q17.Obsessions_1 0.95 0.95 0.99 0.66 21 0.016 0.038 0.67

Q17.Obsessions_2 0.96 0.96 0.99 0.70 25 0.013 0.032 0.70

Q17.Obsessions_3 0.95 0.95 0.99 0.65 20 0.017 0.039 0.66

Q17.Obsessions_4 0.96 0.96 0.99 0.68 23 0.014 0.041 0.70

Q17.Obsessions_5 0.95 0.95 0.99 0.65 20 0.016 0.038 0.66

Q18.Compulsion.frq_1 0.96 0.96 0.99 0.69 25 0.013 0.031 0.71

Q19.Compulsions_1 0.95 0.96 0.99 0.67 22 0.015 0.039 0.70

Q19.Compulsions_2 0.96 0.96 0.99 0.69 25 0.013 0.036 0.70

Q19.Compulsions_3 0.95 0.95 0.99 0.65 20 0.017 0.038 0.66

Q19.Compulsions_4 0.96 0.96 0.99 0.67 23 0.014 0.039 0.69

Q19.Compulsions_5 0.95 0.95 0.99 0.65 21 0.016 0.037 0.67

Item statistics

n raw.r std.r r.cor r.drop mean sd

Q16.Obsession.frq_1 22 0.81 0.78 0.78 0.76 2.1 1.06

Q17.Obsessions_1 22 0.92 0.91 0.91 0.90 2.8 0.68

Q17.Obsessions_2 22 0.66 0.69 0.69 0.61 2.0 0.80

Q17.Obsessions_3 22 0.95 0.94 0.94 0.93 2.2 0.98

Q17.Obsessions_4 22 0.78 0.80 0.80 0.75 1.6 0.67

Q17.Obsessions_5 22 0.94 0.94 0.94 0.93 2.5 0.94

Q18.Compulsion.frq_1 22 0.75 0.72 0.71 0.68 2.1 1.12

Q19.Compulsions_1 22 0.85 0.85 0.84 0.82 2.7 0.78

Q19.Compulsions_2 22 0.68 0.71 0.70 0.63 1.9 0.72

Q19.Compulsions_3 22 0.95 0.94 0.94 0.94 2.2 0.95

Q19.Compulsions_4 22 0.78 0.81 0.81 0.74 1.7 0.79

Q19.Compulsions_5 22 0.94 0.93 0.93 0.93 2.4 0.91

##### **Cronbach Alpha Analysis on Obsessions Questions (average)**

Reliability analysis

Call: psych::alpha(x = participant_means %>% select(all_of(six_items)),

na.rm = TRUE)

raw_alpha std.alpha G6(smc) average_r S/N ase mean sd median_r

0.89 0.92 0.95 0.67 12 0.023 1.9 0.6 0.71

95% confidence boundaries

lower alpha upper

Feldt 0.80 0.89 0.95

Duhachek 0.84 0.89 0.94

Reliability if an item is dropped:

raw_alpha std.alpha G6(smc) average_r S/N alpha se var.r med.r

Q16.Obsession.frq_1 0.92 0.92 0.94 0.71 12.0 0.025 0.023 0.69

Q17.Obsessions_1 0.85 0.90 0.93 0.65 9.2 0.030 0.032 0.69

Q17.Obsessions_2 0.88 0.93 0.94 0.74 14.2 0.019 0.020 0.74

Q17.Obsessions_3 0.84 0.89 0.92 0.62 8.2 0.037 0.033 0.64

Q17.Obsessions_4 0.87 0.92 0.94 0.69 11.3 0.026 0.043 0.74

Q17.Obsessions_5 0.83 0.89 0.92 0.62 8.2 0.037 0.031 0.65

Item statistics

n raw.r std.r r.cor r.drop mean sd

Q16.Obsession.frq_1 22 0.70 0.78 0.74 0.69 0.1 0.053

Q17.Obsessions_1 22 0.88 0.90 0.89 0.83 2.8 0.681

Q17.Obsessions_2 22 0.77 0.72 0.66 0.65 2.0 0.798

Q17.Obsessions_3 22 0.95 0.95 0.96 0.91 2.2 0.983

Q17.Obsessions_4 22 0.82 0.81 0.76 0.74 1.6 0.670

Q17.Obsessions_5 22 0.95 0.95 0.96 0.91 2.5 0.943

##### **Cronbach Alpha Analysis on Compulsions Questions (average)**

Reliability analysis

Call: psych::alpha(x = participant_means_comp %>% select(all_of(six_comp_items)),

na.rm = TRUE)

raw_alpha std.alpha G6(smc) average_r S/N ase mean sd median_r

0.89 0.91 0.94 0.63 10 0.026 1.8 0.61 0.63

95% confidence boundaries

lower alpha upper

Feldt 0.79 0.89 0.95

Duhachek 0.83 0.89 0.94

Reliability if an item is dropped:

raw_alpha std.alpha G6(smc) average_r S/N alpha se var.r med.r

Q18.Compulsion.frq_1 0.92 0.92 0.94 0.69 10.9 0.028 0.025 0.66

Q19.Compulsions_1 0.85 0.89 0.92 0.61 7.8 0.034 0.038 0.66

Q19.Compulsions_2 0.88 0.92 0.93 0.68 10.8 0.023 0.032 0.69

Q19.Compulsions_3 0.83 0.87 0.90 0.57 6.7 0.041 0.032 0.58

Q19.Compulsions_4 0.86 0.90 0.91 0.65 9.5 0.028 0.038 0.65

Q19.Compulsions_5 0.83 0.87 0.91 0.58 6.9 0.038 0.032 0.58

Item statistics

n raw.r std.r r.cor r.drop mean sd

Q18.Compulsion.frq_1 22 0.63 0.72 0.66 0.62 0.1 0.056

Q19.Compulsions_1 22 0.88 0.88 0.86 0.81 2.7 0.781

Q19.Compulsions_2 22 0.75 0.73 0.67 0.64 1.9 0.723

Q19.Compulsions_3 22 0.95 0.95 0.96 0.90 2.2 0.949

Q19.Compulsions_4 22 0.82 0.79 0.76 0.73 1.7 0.788

Q19.Compulsions_5 22 0.93 0.93 0.94 0.88 2.4 0.905

##### **Cronbach Alpha Analysis on All Questions (all days)**

Call: psych::alpha(x = items_subset)

raw_alpha std.alpha G6(smc) average_r S/N ase mean sd median_r

0.94 0.96 0.97 0.65 22 0.0024 1.72 0.91 0.66

95% confidence boundaries

lower alpha upper

Feldt 0.94 0.94 0.95

Duhachek 0.94 0.94 0.95

Reliability if an item is dropped:

raw_alpha std.alpha G6(smc) average_r S/N ase var.r med.r

Q16.Obsession.frq_1 0.95 0.95 0.97 0.64 20 0.0026 0.013 0.64

Q17.Obsessions_1 0.94 0.95 0.97 0.64 20 0.0028 0.014 0.64

Q17.Obsessions_2 0.94 0.96 0.97 0.66 22 0.0025 0.012 0.68

Q17.Obsessions_3 0.93 0.95 0.97 0.64 19 0.0029 0.013 0.64

Q17.Obsessions_4 0.94 0.96 0.97 0.66 22 0.0024 0.013 0.68

Q17.Obsessions_5 0.93 0.95 0.97 0.64 19 0.0029 0.013 0.62

Q18.Compulsion.frq_1 0.95 0.95 0.97 0.65 20 0.0026 0.012 0.64

Q19.Compulsions_1 0.94 0.95 0.97 0.64 20 0.0028 0.013 0.64

Q19.Compulsions_2 0.94 0.95 0.97 0.66 21 0.0025 0.014 0.68

Q19.Compulsions_3 0.93 0.95 0.97 0.64 19 0.0029 0.013 0.60

Q19.Compulsions_4 0.94 0.95 0.97 0.66 21 0.0026 0.014 0.66

Q19.Compulsions_5 0.93 0.95 0.97 0.64 19 0.0029 0.012 0.62

Item statistics

n raw.r std.r r.cor r.drop mean sd

Q16.Obsession.frq_1 1335 0.82 0.86 0.86 0.82 0.41 0.29

Q17.Obsessions_1 1334 0.89 0.89 0.88 0.84 2.53 1.28

Q17.Obsessions_2 1335 0.82 0.80 0.77 0.73 1.81 1.25

Q17.Obsessions_3 1335 0.91 0.91 0.91 0.87 2.01 1.34

Q17.Obsessions_4 1335 0.79 0.79 0.75 0.71 1.50 1.07

Q17.Obsessions_5 1335 0.93 0.93 0.92 0.88 2.24 1.38

Q18.Compulsion.frq_1 1335 0.81 0.86 0.85 0.80 0.41 0.30

Q19.Compulsions_1 1335 0.89 0.89 0.88 0.84 2.44 1.34

Q19.Compulsions_2 1335 0.80 0.80 0.77 0.72 1.64 1.12

Q19.Compulsions_3 1335 0.92 0.91 0.92 0.88 1.99 1.36

Q19.Compulsions_4 1335 0.81 0.81 0.78 0.74 1.51 1.11

Q19.Compulsions_5 1335 0.92 0.92 0.91 0.87 2.16 1.36

##### **Cronbach Alpha Analysis on Obsessions Questions (all days)**

Call: psych::alpha(x = items_subset)

raw_alpha std.alpha G6(smc) average_r S/N ase mean sd median_r

0.90 0.92 0.92 0.67 12 0.0037 1.75 0.94 0.64

95% confidence boundaries

lower alpha upper

Feldt 0.89 0.90 0.91

Duhachek 0.89 0.90 0.90

Reliability if an item is dropped:

raw_alpha std.alpha G6(smc) average_r S/N ase var.r med.r

Q16.Obsession.frq_1 0.91 0.91 0.91 0.67 10 0.0041 0.010 0.62

Q17.Obsessions_1 0.86 0.90 0.90 0.65 9 0.0047 0.012 0.62

Q17.Obsessions_2 0.88 0.92 0.91 0.70 12 0.0037 0.012 0.75

Q17.Obsessions_3 0.86 0.90 0.90 0.64 9 0.0050 0.011 0.62

Q17.Obsessions_4 0.88 0.92 0.92 0.71 12 0.0035 0.012 0.75

Q17.Obsessions_5 0.86 0.90 0.89 0.64 9 0.0053 0.009 0.61

Item statistics

n raw.r std.r r.cor r.drop mean sd

Q16.Obsession.frq_1 1335 0.80 0.84 0.81 0.78 0.41 0.29

Q17.Obsessions_1 1335 0.88 0.88 0.88 0.81 2.53 1.28

Q17.Obsessions_2 1335 0.81 0.79 0.75 0.70 1.81 1.25

Q17.Obsessions_3 1335 0.90 0.90 0.91 0.84 2.01 1.34

Q17.Obsessions_4 1335 0.78 0.78 0.73 0.68 1.50 1.07

Q17.Obsessions_5 1335 0.92 0.91 0.93 0.86 2.24 1.38

##### **Cronbach Alpha Analysis on Compulsions Questions (all days)**

Call: psych::alpha(x = items_subset)

raw_alpha std.alpha G6(smc) average_r S/N ase mean sd median_r

0.90 0.92 0.93 0.67 12 0.0036 1.69 0.95 0.66

95% confidence boundaries

lower alpha upper

Feldt 0.89 0.90 0.91

Duhachek 0.89 0.90 0.91

Reliability if an item is dropped:

raw_alpha std.alpha G6(smc) average_r S/N ase var.r med.r

Q18.Compulsion.frq_1 0.91 0.91 0.91 0.68 11 0.0041 0.010 0.63

Q19.Compulsions_1 0.87 0.90 0.91 0.66 10 0.0049 0.011 0.63

Q19.Compulsions_2 0.89 0.92 0.91 0.70 12 0.0033 0.012 0.73

Q19.Compulsions_3 0.86 0.90 0.90 0.64 9 0.0050 0.010 0.61

Q19.Compulsions_4 0.89 0.92 0.91 0.70 12 0.0035 0.011 0.73

Q19.Compulsions_5 0.86 0.90 0.90 0.64 9 0.0049 0.008 0.63

Item statistics

n raw.r std.r r.cor r.drop mean sd

Q18.Compulsion.frq_1 1335 0.78 0.83 0.80 0.76 0.41 0.30

Q19.Compulsions_1 1335 0.89 0.88 0.88 0.82 2.44 1.34

Q19.Compulsions_2 1335 0.79 0.79 0.74 0.70 1.64 1.12

Q19.Compulsions_3 1335 0.91 0.90 0.92 0.85 1.99 1.36

Q19.Compulsions_4 1335 0.80 0.79 0.76 0.71 1.51 1.11

Q19.Compulsions_5 1335 0.91 0.91 0.92 0.85 2.16 1.36

Confirmatory Factor Analysis Model Results

OCD MODEL RESULTS

Two-Tailed

Estimate S.E. Est./S.E. P-Value

Within Level

OCDW BY

Q1R 0.744 0.071 10.478 0.000

Q2 0.814 0.079 10.318 0.000

Q3 0.716 0.101 7.082 0.000

Q4 0.689 0.095 7.248 0.000

Q5 0.507 0.112 4.527 0.000

Q6 0.815 0.099 8.238 0.000

Q7R 0.747 0.090 8.282 0.000

Q8 0.796 0.090 8.897 0.000

Q9 0.651 0.101 6.424 0.000

Q10 0.697 0.095 7.338 0.000

Q11 0.537 0.115 4.668 0.000

Q12 0.743 0.096 7.716 0.000

Residual Variances

Q2 0.341 0.043 8.013 0.000

Q3 0.446 0.068 6.535 0.000

Q4 0.314 0.042 7.536 0.000

Q5 0.447 0.073 6.133 0.000

Q6 0.317 0.032 9.880 0.000

Q8 0.318 0.047 6.711 0.000

Q9 0.357 0.061 5.832 0.000

Q10 0.346 0.055 6.241 0.000

Q11 0.361 0.074 4.881 0.000

Q12 0.323 0.034 9.612 0.000

Q1R 0.431 0.060 7.234 0.000

Q7R 0.478 0.067 7.164 0.000

New/Additional Parameters

OMEGAW 0.941 0.013 71.086 0.000

HW 0.944 0.012 77.322 0.000

Obsession Only MODEL RESULTS

Two-Tailed

Estimate S.E. Est./S.E. P-Value

Within Level

OBW BY

Q1R 0.743 0.076 9.743 0.000

Q2 0.855 0.082 10.464 0.000

Q3 0.745 0.108 6.913 0.000

Q4 0.743 0.100 7.430 0.000

Q5 0.523 0.115 4.540 0.000

Q6 0.862 0.104 8.297 0.000

Residual Variances

Q2 0.274 0.036 7.526 0.000

Q3 0.403 0.075 5.409 0.000

Q4 0.238 0.042 5.711 0.000

Q5 0.430 0.081 5.320 0.000

Q6 0.238 0.028 8.554 0.000

Q1R 0.432 0.061 7.094 0.000

New/Additional Parameters

OMEGA_OB 0.908 0.021 43.127 0.000

H_OBW 0.919 0.018 50.680 0.000

Compulsion Only MODEL RESULTS

Two-Tailed

Estimate S.E. Est./S.E. P-Value

Within Level

CPW BY

Q7R 0.749 0.092 8.159 0.000

Q8 0.827 0.093 8.909 0.000

Q9 0.676 0.103 6.559 0.000

Q10 0.755 0.093 8.091 0.000

Q11 0.557 0.116 4.786 0.000

Q12 0.800 0.097 8.249 0.000

Residual Variances

Q8 0.268 0.043 6.244 0.000

Q9 0.323 0.070 4.633 0.000

Q10 0.262 0.046 5.744 0.000

Q11 0.338 0.076 4.461 0.000

Q12 0.235 0.032 7.260 0.000

Q7R 0.474 0.067 7.065 0.000

New/Additional Parameters

OMEGA_CP 0.909 0.024 38.609 0.000

H_CPW 0.916 0.022 41.533 0.000
