# Supplementary material for: Validation of a Low-Burden, Once-Daily Obsessive-Compulsive Disorder Measure Over 70 Days: Ecological Momentary Assessment Study
Source: JMIR Form Res. 2026 Mar 30;10:e86471. doi: 10.2196/86471 (PMC13035074; doi:10.2196/86471)
Supplement: Checklist 1 [file formative-v10-e86471-s002.docx]

Adapted STROBE Checklist for Reporting EMA Studies (CREMAS)

| Title |  |  |
| --- | --- | --- |
| 1. Title | Include ecological momentary assessment in title and key words | In title |
| Introduction |  |  |
| 2. Rationale | Briefly introduce the concept of EMA and provide reasons for utilizing EMA for this study or topic of interests | Second paragraph of intro background (pg 1) |
| Methods |  |  |
| 3. Training | Indicate if, and by what methods, training of participants for EMA protocol was used | First paragraph of *Study Design and Procedure* section (pg 3) |
| 4. Technology | Describe what technology, if any, was used. Include the following information: device (eg, mobile phone, portable computer), model (eg, Nexus 4, iPod), operating system (eg, Android, Windows), and EMA program name | First and second paragraphs of *Study Design and Procedure* section (pg 3) |
| 5. Wave Duration | State the number of waves for the study (eg, 2 monitoring periods over the course of 1 year) | Single wave as described in pg 3 |
| 6. Monitoring period | State the number of days each wave of the study lasted, and how many weekdays versus weekend days | 70 days, weekdays + weekends (pg 3) |
| 7. Prompting design | Indicate the prompting strategy used for the study (eg, event-based, interval-based, or a combination of the two). If using interval-based strategy, indicate what type of schedule is used (eg, fixed, random, or hybrid interval) | Random interval- randomly sent between 8 am and 8 pm (pg 3) |
| 8. Prompt Frequency | Intended frequency of prompts per day. Break down by weekdays and weekend days if applicable | Once per day, weekdays + weekends were not different (pg 3) |
| 9. Design features | Describe any design feature to address potential sources of bias (eg, reactivity) or participant burden (eg, EMA questions appearing in different orders) | Once-daily nature and allotted 3 hours to complete EMA reduce burden (pg 3) |
| Results |  |  |
| 10. Attrition | Indicate participant attrition throughout the study; report attrition rates both by monitoring days and waves, if applicable | *Compliance* section. 3/25 OCD participants and 2/22 healthy participants withdrawn. (pg 6) |
| 11. Prompt delivery | Report number of EMA prompts that were planned to be delivered. If possible, also report the number of EMA prompts that were actually received by participants and indicate reasons for why prompts were not sent out (eg, technical issues or participant noncompliance reason such as phone was powered off) | 70 prompts were sent for each participant. (pg 3) |
| 12. Latency | Report the amount of time from prompt signal to answering of prompt | *Compliance* section— median of 143 seconds to complete survey (pg 7) |
| 13. Compliance rate | Report total answered EMA prompts across all subjects and the average number of EMA prompts answered per person. Report compliance rate both by monitoring days and waves, if applicable. Indicate reasons for noncompliance, if known | *Compliance* section (pgs 6-7) and Fig. 1 |
| 14. Missing data | Report whether EMA compliance is related to demographic or time-varying variables | *Compliance* section— baseline Y-BOCS did not correlate significantly with EMA compliance rate (pg 7) |
| Discussion |  |  |
| 15. Limitations | Discuss limitations of the study, taking into account sources of potential bias when using EMA methods (eg, reactivity, use of technology) | *Limitations* as part of Discussion (pg 11) |
| 16. Conclusions | Provide a general interpretation of results and discuss the benefits of using EMA | *Conclusions* as part of Discussion (pg 11) |
